# Characterization, Codon Usage Pattern and Phylogenetic Implications of the Waterlily Aphid *Rhopalosiphum nymphaeae* (Hemiptera: Aphididae) Mitochondrial Genome

**DOI:** 10.3390/ijms252111336

**Published:** 2024-10-22

**Authors:** Aiyang Shi, Chenyang Li, Muhammad Farhan, Chunhao Xu, Yanjin Zhang, Hongye Qian, Shuai Zhang, Tianxing Jing

**Affiliations:** College of Plant Protection, Yangzhou University, Yangzhou 225009, China; 19952992581@163.com (A.S.); lcy826lcy@163.com (C.L.); farhan.entomology@gmail.com (M.F.); 18752518708@163.com (C.X.); m15305162061@163.com (Y.Z.); 18068910012@163.com (H.Q.); shuaizhang@yzu.edu.cn (S.Z.)

**Keywords:** mitochondrial genome, *Rhopalosiphum nymphaeae*, codon usage, phylogeny

## Abstract

The water lily aphid, *Rhopalosiphum nymphaeae*, is the only known aphid that can live in both terrestrial and aquatic conditions. In this study, the complete mitochondrial genome of *R. nymphaeae* was generated using Illumina sequencing technology. The typical circular DNA mitochondrial genome of *R. nymphaeae* is 15,772 bp in length, with a high A+T content (84.34%). It contains 37 coding genes (13 protein-coding genes, 22 transport RNAs, and two ribosomal RNAs) and two non-coding regions (one control region and one repeat region). Enc-plot, PR2-bias, and neutrality plot analysis indicated that the codon usage of the protein-coding genes is mainly affected by natural selection. The evolution rate analysis (the ratio of nonsynonymous to synonymous, Ka/Ks) indicated that all the PCGs in *R. nymphaeae* are under a strong purifying selection. The control region has conserved structure elements, and two types of tandem repeat units exist. The length and sequence of the aphid-unique repeat region has high similarity with closely related species. Phylogenetic analyses determined by both maximum likelihood and Bayesian inference support the monophyly of Aphidinae, Aphidini, Aphidina, and Rhopalosiphina. However, the monophyly of the genera in Rhopalosiphina, such as *Rhopalosiphum*, is still not resolved. This study may help us to understand the phylogenetic relationship of aphids, and much more aphid data are needed in future studies.

## 1. Introduction

The water lily aphid, *Rhopalosiphum nymphaeae*, belonging to Aphididae (Hemiptera), is a typical holocyclic polyphagous heteroecious pest aphid. Colonies of this aphid often occur on various water plants, such as *Callitriche*, *Euryale*, *Lemna*, *Nelumbo* and *Nymphaea* [1]. This aphid sucks the plant sap, resulting in leaf rolling. Also, they can secrete honeydew, resulting in lower photosynthetic activity. Furthermore, it is a vector of virus transmission on aquatic vegetables [2]. Thus, this aphid is considered an important pest on aquatic vegetables such as water chestnuts and water lilies in China and India [1]. It was reported that aquatic vegetable pests, including *R. nymphaeae*, can cause yield loss between 17.04 and 23.66% [1]. Otherwise, it has been used as an enemy to control water weeds in America [3]. *R. nymphaeae* has some ability to survive under water, and it is the only known aphid that can live in both terrestrial and aquatic conditions [4]. Its host range is quite different compared to other *Rhopalosiphum* aphid species. The host alternation of aphids is an unusual characteristic in the animal kingdom and the phylogeny of aphids is usually based on morphological characters, molecule data and host information [5]. Rhopalosiphina is one monophyletic group in Aphidini. However, the relationship in Rhopalosiphina, such as the *Rhopalosiphum* genus, is still not resolved [5,6]. With the special host range and aquatic habitat, *R. nymphaeae* may give us some clues.

For most insects, the mitochondrial genome DNA molecule is double-stranded and circular. It mainly contains one non-coding region (control region), two rRNAs (ribosomal RNA genes), 13 PCGs (protein-coding genes) and 22 tRNAs (transfer RNA genes). In Aphidinae aphids, one more unique non-coding region (repeat region) exists in the mitochondrial genome [7,8,9]. Mitochondrial genes are commonly used and powerful molecular markers in species identification, phylogenetic relationship and population structure because of their small molecular mass, maternal inheritance and fast evolutionary rate [7,10]. For instance, phylogenetic analysis based on mitochondrial genomes determined the relationship between two genera, *Toxoptera* and *Aphis* [8], and a biogeography study based on mitochondrial genes indicated that Aphidini may be European in origin [5]. To date, only three mitochondrial genomes of *Rhopalosiphum* species, including the incomplete mitochondrial genome of *R. padi*, are available.

In this study, we obtained the complete mitochondrial genome of *R. nymphaeae* by employing high-throughput sequencing technique. Overall, the size, nucleotide composition, organization, codon usage and bias, evolution rate, and non-coding regions of *R. nymphaeae* were analyzed and described. The phylogenetic relationships of *R. nymphaeae* with other aphids were constructed based on 13 PCGs and two rRNAs. Our results may help studies of the phylogenetic relationships and population genetics of this species, as well as studies on the *Rhopalosiphum* genus in the future.

## 2. Results and Discussion

### 2.1. Characterization of Mitochondrial Genome

**Figure 1:**
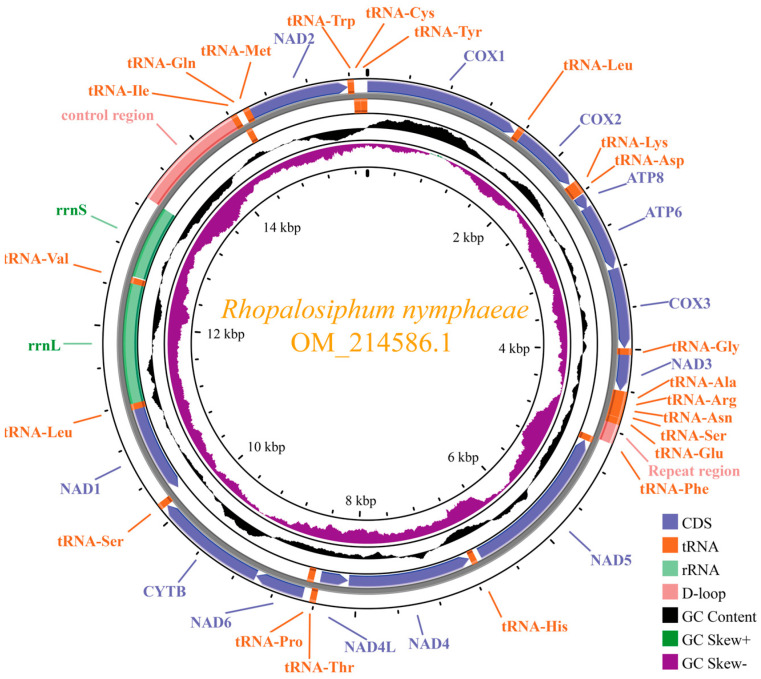
Circular maps of the mitogenomes of *Rhopalosiphum nymphaeae*.

The complete *R. nymphaeae* mitochondrial genome (GenBank accession number: OM214586.1, https://www.ncbi.nlm.nih.gov/, accessed on 11 January 2022) is a typical circular double-strand structure with a length of 15,772 bp (Figure 1). The size of the *R. nymphaeae* mitogenome in this study is larger than two previously reported *R. nymphaeae* mitogenomes collected from Republic of Korea and Hunan province of China (NC_046740.1 and MZ420705.1) because of the differences in the length of the AT-rich region (control region) [11]. This *R. nymphaeae* mitochondrial genome contains 37 coding genes, including 2 rRNAs (ribosomal RNAs), 13 PCGs (protein-coding genes) and 22 tRNAs (transport RNAs) (Table 1). Two long non-coding regions are found in the *R. nymphaeae* mitogenome. The larger non-coding region (1109 bp) is situated between rrnS and tRNA-Ile, and this region is usually known as the control region [9,12,13]. Another non-coding region (193 bp) is located between two tRNAs (Glu and Phe). This shorter region, often mentioned as a “repeat region”, has been considered a unique feature of aphid mitochondrial genomes [12,14]. The gene arrangement of the *R. nymphaeae* mitogenome is the same as that of most aphids, such as *Aphis gossypii* and *A. glycines* [9,15,16,17]. Thirteen overlapping regions (ranging from 1 to 20 bp) and thirteen intergenic spacers (ranging from 1 to 52 bp) exist in the *R. nymphaeae* mitogenome. The most prominent intergenic spacer is located between NAD5 and tRNA-His, and the largest overlapping region is situated between ATP8 and ATP6. The locations of these two largest intergenic spacer/overlapping regions are conserved in aphids [8,10,14,18].

In the mitogenome, DNA strand asymmetry is prevalent, and nucleotides are usually distributed differentially between the two DNA strands [19]. The minority strand (- strand) encodes four NADH dehydrogenase complex genes (NAD1, NAD4, NAD4L and NAD5) and eight tRNA genes (tRNA-Phe, His, Pro, Leu1, Val, Gln, Cys and Tyr), while the majority strand (+ strand) encodes the other nine PCGs and fourteen tRNA genes. The nucleotide composition of *R. nymphaeae* mitogenome is shown in Table 2. The whole mitogenome shows a bias toward the AT content, with the nucleotide frequencies of A:44.98%, T:39.36%, G:5.78% and C:9.88%, respectively. For individual genes (PCGs, tRNAs, rRNAs) and non-coding sequences, the A+T bias are also obvious. This is similar to other aphids’ mitogenome, with the AT content ranging from 82.2% to 85.4% [8,9,12]. Like other aphid species, the *R. nymphaeae* mitogenome full-length presents as slightly A-skewed, with a positive AT-skew value of (0.0067) and strongly C-skewed, with a negative GC-skew value of (−0.2618) [8,18]. Negative values of AT-skew and GC-skew exist in PCGs and the + strand (the majority of PCGs located on the + strand). The asymmetry feature is a common phenomenon in the mitochondrial genome and is influenced by a series of factors, such as mutation, mitochondrial replication and transcription [19,20].

### 2.2. PCGs, Relative Synonymous Codon Usage Analysis

The thirteen PCGs of *R. nymphaeae* were 10,943 bp total in size, and they encoded 3636 amino acids. The PCG length, start, and stop codons are shown in Table 1. NAD5 was the longest PCG with 1671 bp, and ATP8 was the shortest PCG with 159 bp. All the PCGs initiated with the ATN start codon, including three ATGs, four ATAs, and six ATTs. Correspondingly, most PCGs terminated with the usual TAG or TAA stop codons, except COX1 and NAD4. At the 3′ end of the COX1 and NAD4 genes, the single “T” base is the signal of termination and can be completed by polyadenylation after transcription [21]. Such an incomplete stop codon T is common in insects [22,23]. Although in several aphids, such as *A. citricidus*, TAA was found at the ending of COX1 and NAD4, it was uncertain whether TAA was recognized as a true codon or T was matured to a complete stop codon by post-transcriptional polyadenylation [21].

**Figure 2:**
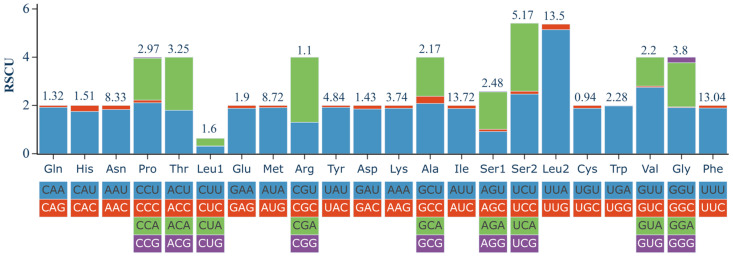
Relative synonymous codon usage (RSCU) of the *Rhopalosiphum nymphaeae* mitochondrial genome. The amino acid frequencies are marked above the bars.

The codon usage of PCGs in the *R. nymphaeae* mitogenome was shown by RSCU (Figure 2). Among the 20 amino acids, Ile (13.72%), Leu2 (13.5%) and Phe (13.04%) were the three most frequently used amino acids, while Cys (0.94%) was the least used amino acid. In total, 54 codons were used in the *R. nymphaeae* mitogenome PCGs. Four codons translated three amino acids (Gly, Pro, Ser1); four amino acids (Ala, Leu1, Ser2 and Val) were translated by three codons; and two codons translated the remaining fifteen amino acids. The RSCU value of 28 codons was greater than 1 (1.2–5.14) and the third bases of all these 28 high-frequency codons were A or U. The high A and U bases in the codons contributed to the strong AT bias in PCGs. The three most abundant codons were UUA (Leu2), UCA (Ser2) and GUU (Val), while six codons (GCG-Ala, CGG-Arg, CUG-Leu1, UCG-Ser2, ACG-Thr and GUG-Val) were not present in the PCGs.

### 2.3. Enc-plot, PR2-bias and Neutrality Plot Analysis

**Figure 3:**
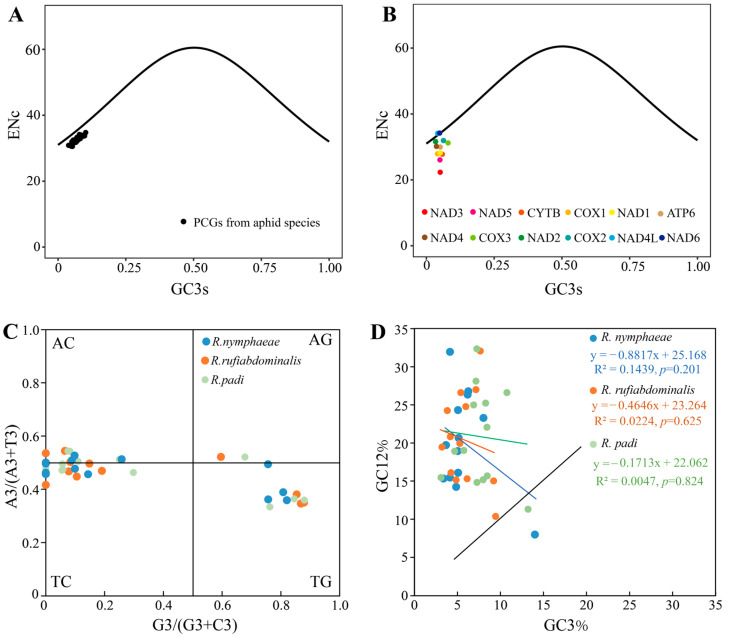
Enc-plot, Parity Rule 2 bias, and Neutrality plot analysis of *Rhopalosiphum nymphaeae* mitochondrial genome. (**A**) Enc-plot analysis of concatenate of 13 PCGs from 17 Aphidinae species; (**B**) Enc-plot analysis of individual PCGs of *R. nymphaeae*; (**C**) Parity Rule 2 (PR2) bias of three *Rhopalosiphum* species; (**D**) Neutrality plots analysis of three *Rhopalosiphum* species, the diagonal line was shown in black line.

The Enc (effective number of codons) value has been extensively used to estimate the codon bias level of PCGs. The Enc values of PCGs (concatenate of 13 PCGs) from 17 Aphidinae species and 12 individual PCGs (except ATP8, because of its short length) from *R. nymphaeae* were calculated (Figure 3A,B). The lowest Enc value of the concatenate sequences was found in *Acyrthosiphon pisum* (30.59) and the highest Enc value was found in *Pterocomma pilosum* (33.63). The Enc values of individual *R. nymphaeae* PCGs ranged from 22.31 (NAD3) to 34.20 (NAD6). The theoretical Enc value interval ranged from 20 to 61, and the lower (<35) ENc values reflected the higher level of codon usage bias [24]. The Enc values of all the test PCGs were less than 35, indicating that all these PCGs had a high codon usage bias level. The ENc-plot showed that all the concatenate PCG sequences in Aphidinae were located below the standard curve line, revealing that not only mutational pressure but also other pressures, such as natural selection pressure, influenced these PCGs [24,25]. For individual PCGs in *R. nymphaeae*, two genes, NAD6 and NAD4L, were laid on the expected curve, indicating that only mutational pressure acted on these two genes. The other ten PCGs were located below the curve, especially the NAD3 gene, which means the codon bias of these PCGs was mainly determined by natural selection and other factors [25,26].

PR2 (Parity Rule 2)-bias plot analysis was performed based on the third codon base contents of 13 individual PCGs in three *Rhopalosiphum* aphids (*R. nymphaeae*, *R. rufiabdominalis* and *R. paid*). The axes were centered on 0.5 and divided into four quadrants. The central point (0.5, 0.5) indicates that only mutation pressure leads to codon bias [25,27,28]. Most PCGs in the *Rhopalosiphum* species were in the third and the second quadrants, while no PCG of *R. nymphaeae* was in the first quadrant (Figure 3C). This result means that the U content of the third codon was higher than A and the C was more frequent than G. Generally, the A and U base contents, as the G and C base contents, should be equal in the third codon. However, PR2 bias analysis of the *Rhopalosiphum* species provided evidence that not only mutation pressure but some other factors, maybe natural selection, influenced the codon bias. Notably, four PCGs (NAD3, NAD6, COX1 and COX2,) in *R. nymphaeae*, three PCGs (ATP8, COX2 and NAD6) in *R. rufiabdominalis* and one PCG (ATP8) in *R. padi* had no G base on the third codon.

Neutrality plots are drawn in Figure 3D to display the relationship between GC12 and GC3. In *R. nymphaeae*, the GC12 content of the individual PCG varied from 8.00% (ATP8) to 31.96% (COX1) and the GC3 content ranged from 3.37% (NAD2) to 14.00% (ATP8). The contents of GC12 and GC3 were weakly correlated with a regression coefficient of −0.88 (R^2^ = 0.1439, *p* = 0.201). There was no significant correlation between the GC12 and GC3 content, which means that mutation pressure has minor effect on the codon bias in *R. nymphaeae*. All the mitochondrial PCGs in three *Rhopalosiphum* species were not laid on the black line (diagonal distribution). Together with the narrow distribution of the GC3 values, our results indicated that GC12 and GC3 were not in the mutation bias model doubtlessly [29,30]. A weak correlation between GC12 and GC3 was also observed between the other two *Rhopalosiphum* species. These results are consistent with most other species’ mitochondrial genomes, which were considered affected dominantly by natural selection [31].

### 2.4. Nucleotide Diversity and Evolution Rate

**Figure 4:**
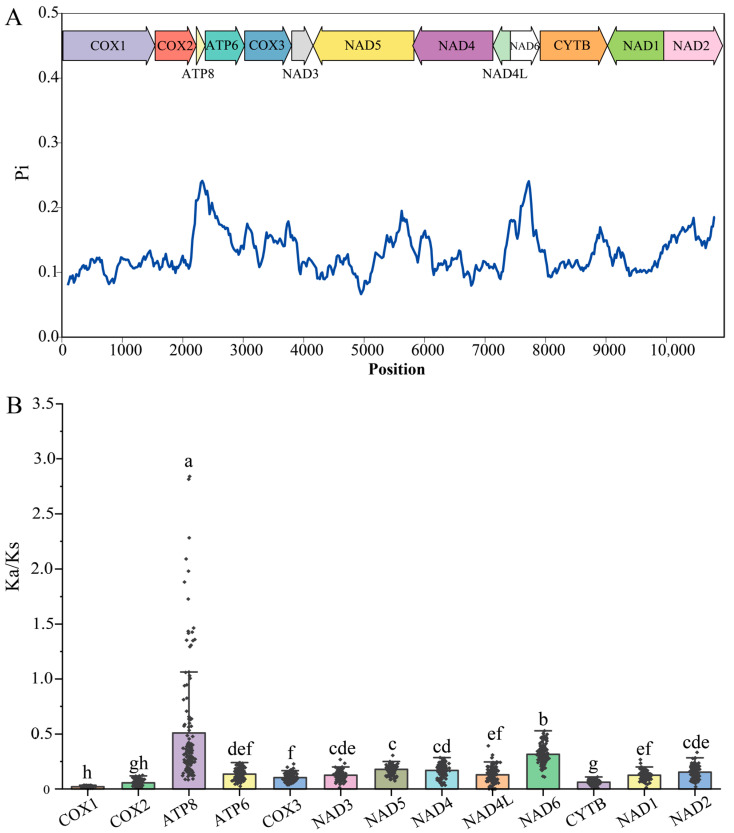
(**A**) Nucleotide diversity of 13 PCGs in aphid mitochondrial genomes. (**B**) Evolutionary rates (the ratio of nonsynonymous to synonymous, Ka/Ks) of 13 mitochondrial PCGs among 17 Aphidinae species. Ka/Ks values were shown as black dots, and different letters above the columns indicate a significant difference of Ka/Ks values among 13 PCGs (mean ± SEM, *p* < 0.05).

The nucleotide diversity (Pi) for the PCGs of 36 aphid species is shown in Figure 4A. Sliding window analysis pointed out that different genes have different Pi values. Among 36 aphid species, the Pi (average value) value of the individual PCGs ranged from 0.108 to 0.228. The ATP8 gene had the highest Pi value with 0.228, followed by NAD6 (0.182) and ATP6 (0.168). Consistent with other studies, the COX1 gene displayed the lowest variability (0.108) [32]. This means that COX1 has high genetic stability among aphids. The nucleotide diversity is commonly used as a guideline for selecting potential gene species-specific markers to study population genetics [33,34]. The nucleotide substitutions, including synonymous sites (Ks) and nonsynonymous sites (Ka), of each PCGs from 17 Aphidinae species were obtained using DNAsp v6 software. For each of the 13 PCGs, the Ka and Ks values between different species are listed in Table S1. Based on the paired samples t-test, the Ka values were significantly lower (*p* < 0.001) than the Ks values for each PCG (Figure S1). This means the Ka/Ks values were <1. Among 17 Aphidinae species, the average Ka/Ks of each PCG ranged from 0.02 to 0.51 (Figure 4B). The lower Ka/Ks values (< 1) indicated the predominant role of purifying selection in all the mitochondrial PCGs in the Aphidinae species. The ATP8 has the highest evolution rate, with an average Ka/Ks value of 0.51, followed by NAD6 (0.316). The relatively highest evolutionary rates on ATP8 and some NADH dehydrogenase complex genes were commonly observed in aphids [7,12]. For most Aphidinae species, the Ka/Ks values of ATP8 are less than 1, but in Figure 4B, we observe several values greater than 1. These greater Ka/Ks values are the ATP8 gene of *Schizaphis graminum* versus other aphids’ ATP8s. The sequence of *S. graminum* was different from other aphids (Figure S2). Most ATP8 genes start to be translated at the common initiation site (ATAGCACCA). Still, the translation initiation site of ATP8 (ATATCACTT) in *S. graminum* was upstream (19 bp) of this common initiation site because of one base deletion in ATP8. This deletion caused translation termination when starting at the common initiation site. When starting at the new upstream initiation site, translation worked normally. However, the translation product was quite different from other aphids. This deletion has also been observed in *Hormaphis betulae* (NC_029495.1). This ATP8 from two aphids was obtained by Sanger dideoxy sequencing with high reliability, but we cannot rule out the possibility that the sequence was incorrect. In contrast, COX1 (0.02) has the lowest evolution rate and appears to have experienced strong purifying selection. In insects, the COX1 gene is the most frequently sequenced and has been used as a molecular marker for evolutionary studies because of its suitable level of variability [35,36]. Analogously, COX2 and CYTB also have lower nucleotide diversity and evolution rates in aphids, which suggests they may also be candidate markers.

### 2.5. Transfer and Ribosomal RNAs

**Figure 5:**
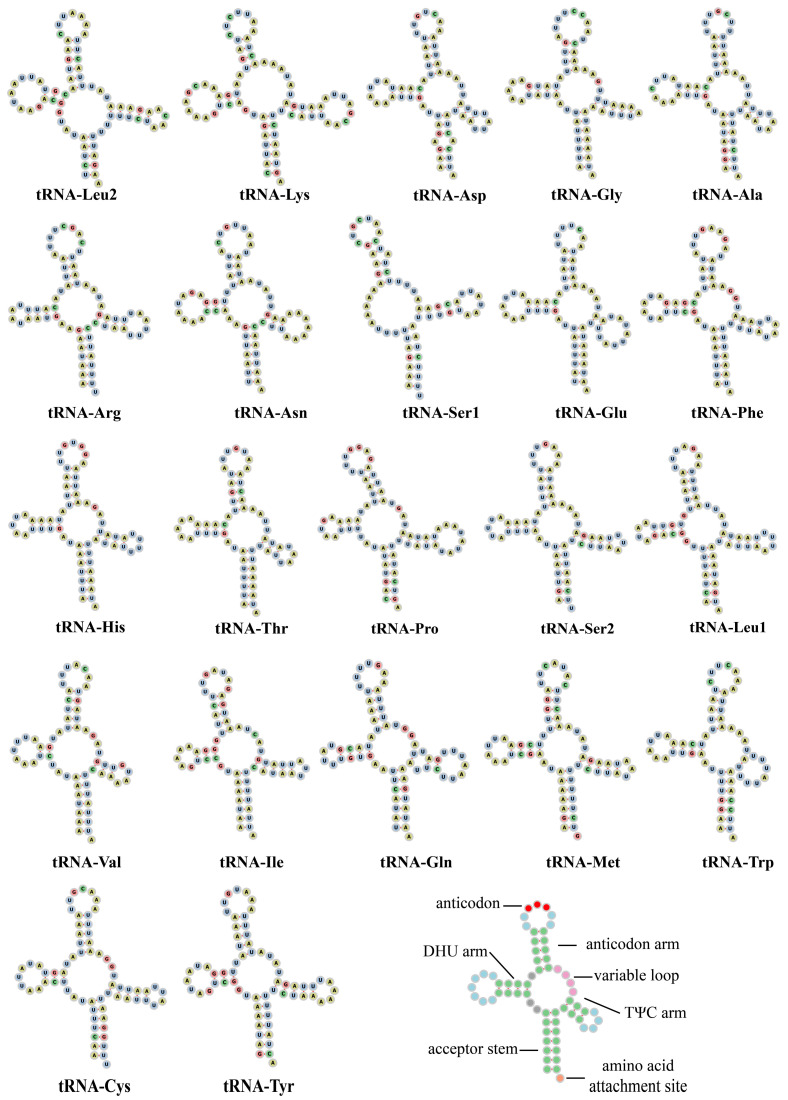
Predicted secondary structures of 22 tRNAs in *Rhopalosiphum nymphaeae*.

The length of the 22 tRNA genes of *R. nymphaeae* varies from 62 bp (tRNA-Ser1, tRNA-Thr, tRNA-Val and tRNA-Trp) to 73 bp (tRNA-Lys). Two tRNAs (tRNA-Ser and tRNA-Leu) have two copies with differences in their anticodons. These two copies are usually present in aphid mitochondrial genomes [7,12,14,18]. Twenty-one tRNAs, except the tRNA-Ser1 (AGN), can be folded into the classical secondary structure, clover-leaves (Figure 5). In most aphids, as well as in other metazoans, the DHU arm (dihydrouridine arm) of tRNA-Ser1 is usually absent [7,12,37].

Two rRNAs, rrnL (16S) and rrnS (12S), are encoded on the—strand. The length and location of these two rRNA genes are conserved in aphids. The rrnL (1256 bp) is larger than rrnS (767 bp), and it also shows a higher A+T content (85.19%) that rrnS (83.70%). The rrnL is located between two tRNA genes (Leu1 and Val), while the rrnS is located between a tRNA gene (Val) and the control region.

### 2.6. Control Region and Repeat Region

**Figure 6:**
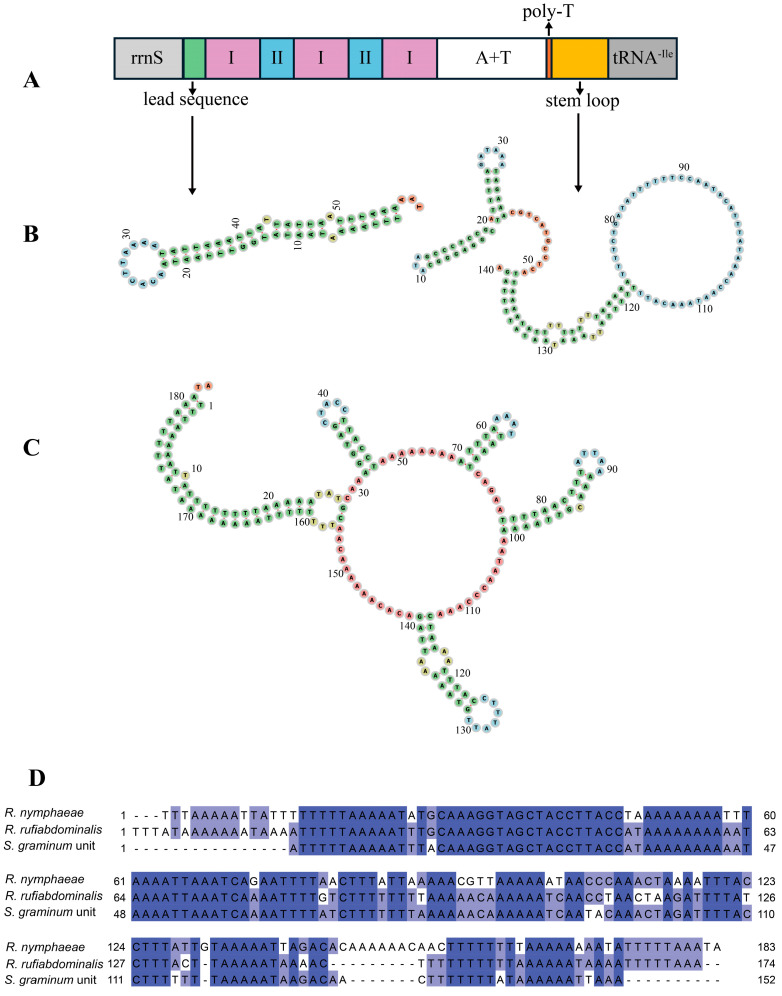
(**A**) The lead sequence is shown in the green box; the purple and blue boxes with Roman numerals indicate the tandem repeat units I and II, respectively; the A+T rich region is shown in the white box; the poly-thymidine stretch is shown in the orange box; the yellow box indicates the steam-loop region. (**B**) The secondary structure of the lead sequence and the stem-loop region. (**C**) The secondary structure of the repeat region. (**D**) The sequence alignment among the entire length of the *R. nymphaeae* repeat region, the full length of *R. rufiabdominalis* repeat region and the repeat unit sequence of *S. graminum.* Numbers showed the base positions from 5′ to 3′.

The non-coding control region is 1109 bp in length and has a high A+T content level (88.09%). It is located between rrnS and tRNA-Ile and it can be divided into five parts: a 58 bp-lead region, which can fold into a stem-loop and be considered involved in the replication and transcription of mitochondrial DNA [38,39,40]; a tandem repeat region, which contains three type I (142 bp) repeat units and two type II (93 bp) repeat units (I-II-I-II-I); a 286-bp region with high A+T content (87.76%); a conserved poly-thymidine stretch; and a 140 bp-stem-loop region (Figure 6A). The secondary structures of the lead sequence and the stem-loop region are shown in Figure 6B. Mitochondrial genomes of *R. nymphaeae* collected from Republic of Korea and Hunan province (China) were sequenced before, but their length was shorter [11]. The length difference existed in the control region. Other parts of these three mitochondrial genomes, such as the repeat region, rRNAs and PCGs, were conserved in length and sequence similarity. The lead region and tandem repeat (unit I-II-I-II) were absent and only a single unit II existed in the control region (650 bp) of the Hunan colony’s genome. For the control region (869 bp) of the Korea colony genome, the lead region was lost, and only two unit I and one unit II (I-II-I) were found (Figure S3).

In aphid mitochondrial genomes, another unique long non-coding region was usually found between two tRNAs (tRNA-Phe and -Glu). This non-coding region with high AT content was regarded as another origin of replication [7,12]. It mainly contained numerous tandem repeats and was consistently named a “repeat region”. However, the repeat region exhibits poor conservation in length, and it is not possible to find tandem repeats in all aphids’ repeat regions [10,14]. In *R. nymphaeae*, the location of the repeat region was conserved, with a size of 193 bp and an A+T content of 86.01%. In already reported aphid mitochondrial genomes, the size of the repeat regions ranged from 179 bp (*A. craccivora*) to 2322 bp (*A. glycines*) [9,41]. In most of these aphids, tandem repeats were found in their repeat regions, such as *S. graminum*, which had four 152 bp tandem repeats and *Ac. pisum* had seven 205 bp tandem repeats [12]. However, *R. nymphaeae* did not have any SSRs or long repeats (>30 bp) but had several short complementary repeats, and these repeats could be folded into stem-loop structures (Figure 6C, Tables S2 and S3). The total length of the repeat region in *R. nymphaeae* was approximately equal to the length of tandem repeat units in other aphids. Previous studies found that tandem repeat units exhibit high sequence similarity among closely related species [7,8,9]. Due to the lack of *R. padi* repeat region information in GenBank, we aligned the full length of the repeat region of *R. nymphaeae* with the full repeat region of *R. rufiabdominalis* and the repeat unit of *S. graminum*. Repeat regions may originate after speciation events of aphids and subsequently experience numerous losses or amplifications during species diversification [8,9]. The full-length repeat region of *R. nymphaeae* shared high similarity with the repeat unit of *S. graminum* (Figure 6D), indicating that the *R. nymphaeae* repeat region may experience a loss of other repeat copies and retain only one copy of the repeat unit.

### 2.7. Phylogenetic Analysis

**Figure 7:**
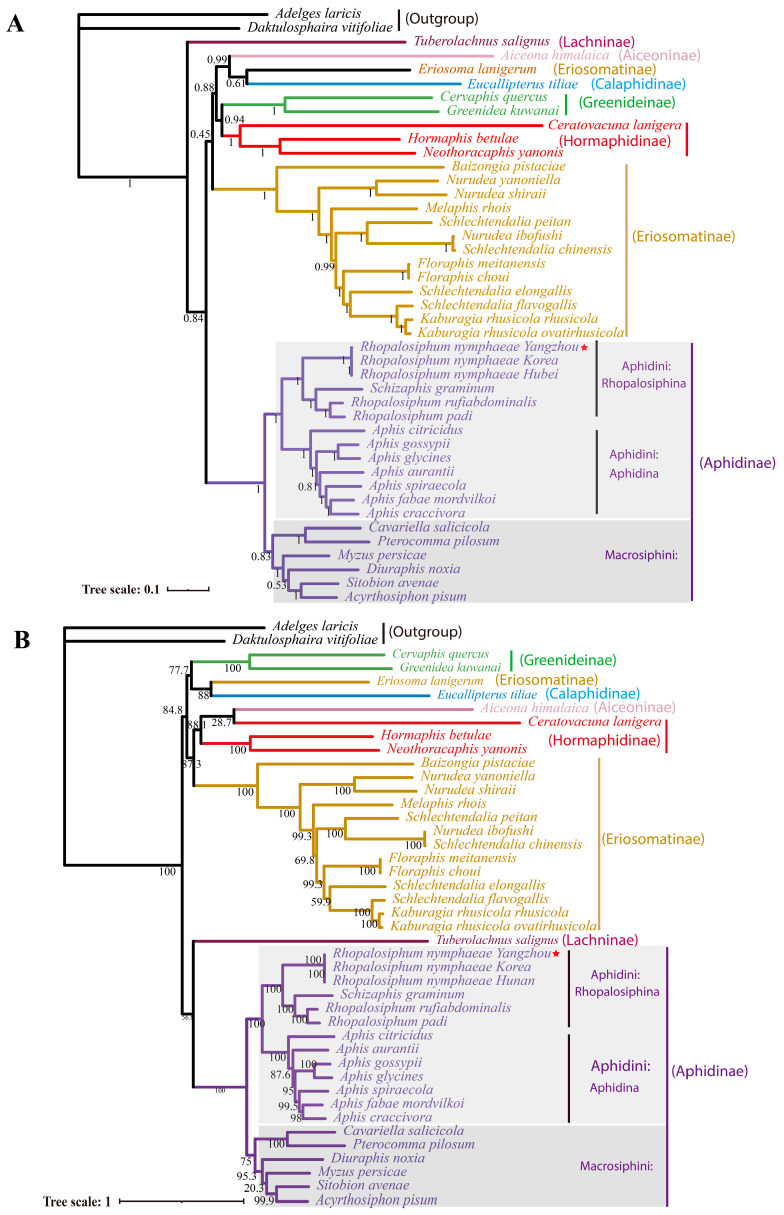
Phylogeny of aphids inferred from the mitochondrial genomes. (**A**) Bayesian tree. The values at node indicate posterior probabilities. (**B**) Maximum-likelihood tree. The values at the nodes indicate bootstrap probabilities. Tree scale is shown at the lower-left conner. *Rhopalosiphum nymphaeae* mitochondrial genome in this study was marked with a red star.

The concatenated dataset (13 PCGs + 2 rRNA) of 41 aphids was used to determine the phylogenetic relationship among the aphids. The Bayesian (Figure 7A) and Maximum-likelihood trees (Figure 7B) almost had a consensus topology in Aphidinae. Consistent with the topological structure constructed by other studies using complete mitogenome sequences or other datasets (tRNA+COX2), both the Maximum-likelihood and Bayesian trees supported the monophyly of Aphidinae. The monophyly of Aphidinae was not only supported by molecular data but also by morphology [42]. Within Aphidinae, both two tribes, Macrosiphini and Aphidini, were found to be monophyletic. In Aphidini, consisting of previous molecular data, morphological studies and ecological research, both the ML and BI results supported the monophyly of Aphidina and Rhopalosiphina [7,43,44]. In both Figure 7A,B, the Rhopalosiphina clade included two genera, *Rhopalosiphum* and *Schizaphis*. *R. padi* and *R. rufiabdominalis* were clustered together, and they were relatively closer to *S. graminum* than to *R. nymphaeae*, the species from the same genus. This means that the genus *Rhopalosiphum* was not a monophyletic group, which was consistent with previous studies [5,42,43,45]. The hosts of *R*. *padi*, *R. rufiabdominalis* and *S. graminum* were similar, and they are mainly Gramineae plants, such as *Triticum aestivum* and *Zea mays*. The phylogenetic relationship based on mitochondrial data was more similar to the aphid host plant association rather than the morphology. Many researchers stated that the inconsistencies between the phylogenetic and taxonomic relationships are likely caused by faulty diagnoses for the genera in Rhopalosiphina. Thus, further works on morphology, molecular and ecological data are needed to revise the generic division and classification within Rhopalosiphina [6].

## 3. Materials and Methods

### 3.1. Aphid Collection, DNA Extraction and Mitochondrial Genome Sequencing

*Rhopalosiphum nymphaeae* was collected from lotus leaves in Yangzhou city (32°23′20″ N, 119°25′12″ E), Jiangsu Province, China. The specimen was identified according to its external morphological characters and then frozen in liquid nitrogen immediately. More than two hundred aphids were ground in liquid nitrogen. Then, the DNeasy Blood and Tissue Kit (Qiagen, Hilden, Germany) was used to extract the total genomic DNA of *R. nymphaeae*, referring to the instructions. The 860 bp COX1 sequence was amplified and sequenced (Sanger sequencing) by a pair of universal primers (forward primer: ACTAATCATAAAGATATTGGAA; reverse primer: CCAATTGTAAATATATGATG).

Before sequencing, the integrity and quantity of the genome DNA were detected by using gel electrophoresis (Liuyi Biotechnology, Beijing, China) and NanoDrop 2000 (Thermo Fisher Scientific, Waltham, MA, USA). Then, the DNA library (∼300 bp fragment) was constructed by PCR amplification and the size selection according to the manufacturer’s instructions for the Agencourt AMPure XP-PCR Purification Beads and Agencourt SPRIselect Beads (Beckman Coulter, Brea, CA, USA). The DNA library was sequenced with the aid of the Illumina Hiseq 2500 platform (2 × 150 bp paired-end reads) at Genesky Biotechnologies Inc., (Shanghai, China). Finally, 879,448 clean reads with 131,320,557 clean bases were obtained.

### 3.2. Assembly and Annotation of Rhopalosiphum nymphaeae

The quality reads were assembled to generate contigs using metaSPAdes software (v 3.13.0, Saint Petersburg, Russia) and then assembled to obtain the *R. nymphaeae* mitochondrial genome using MitoMaker software (v1.14, Rio de Janeiro, Brazil). The assembled mitochondrial genome sequence was submitted to GenBank with the accession number OM214586.1 (https://www.ncbi.nlm.nih.gov/nuccore/OM214586.1/, accessed on 11 January 2022). The complete mitochondrial genome of *Aphis glycines* was set as the reference genome. The assembled genome sequence of *R. nymphaeae* was submitted to the MITOS web server (http://mitos2.bioinf.uni-leipzig.de/index.py, accessed on 2 July 2024) to annotate the PCGs and rRNAs. The location and secondary structure of the tRNAs were verified by both MITOS and tRNAscan-SE 2.0 (http://lowelab.ucsc.edu/tRNAscan-SE/, accessed on 2 July 2024), and their secondary structures were drawn by online TBI-forna tool (http://rna.tbi.univie.ac.at/forna/, accessed on 27 July 2024). BLAST validated the annotation mitochondrial genome sequence on NCBI, and the intergenic spacer and overlapping regions were manually analyzed. The circular map of *R. nymphaeae* was generated using the free online server CGView (http://stothard.afns.ualberta.ca/cgview_server/, accessed on 2 July 2024).

### 3.3. Analysis of Codon Usage Bias

The base compositions of the whole mitochondrial genome and individual regions in *R. nymohaeae*, such as, PCGs, rRNAs, control region etc., were calculated using MEGA X [46], and the RSCU (relative synonymous codon usage) of the PCGs was calculated and drawn by PhyloSuite [47,48]. The following formula calculated the GC skew and AT skew: AT skew =(A−T)/(A+T), GC skew = (G−C)/(G+C).

The CodonW program downloaded from (http://codonw.sourceforge.net, 9 July 2024) and CUSP (EMBOSS Explorer (bioinformatics.nl)) were used to obtain a series of codon usage bias indices, including the Enc value (effective number of codons), GC3 value (G+C content at the third positions of codons), etc. The pattern of codon usage for each PCG in *R. nymphaeae* was evaluated by the ENc-plot individually. The ENc values were set as the ordinate, while the GC3 values were set as the abscissa. The formula calculates the expected curve of ENc: ENcexp = 2 + GC3s + 29/[GC3s^2^ + (1 − GC3s)^2^] [24]. The Enc-plot was generated using the R programming language in RStudio v4.3.3 software and the R script can be found in Supplementary script 1. Also, 13 PCGs from all 41 aphid species were concatenated, and these concatenated sequences were used to perform the ENc-plot evaluation.

The PR2 (Parity Rule 2) bias was detected by the degree of AT and GC-bias. The value of [A3/(A3+ T3)] was set as the ordinate (AT-bias) and the value of [G3/(G3 + C3)] was set as the abscissa (GC-bias) in Excel [28]. A3, G3, C3 and T3 denoted the A, G, C, and T content in the third codon position.

The neutrality plot analysis of the 13 PCGs in *R. nymphaeae* was conducted to examine the relationship between GC12 and GC3. The GC12 values were set as the ordinate, while the GC3 values were set as the abscissa. The linear relationship between the GC3 variables and the GC12 variables was estimated using Excel. The evolution rate (Ka/Ks) of each PCG was calculated using DnsSP v6.12.03 with the genetic code of the invertebrate mitochondrial genome [49]. The Ka and Ks values were compared by paired sample t-test in SPSS, and the Ka/Ks values among the 13 PCGs were analyzed by one-way ANOVA, and the mean values were separated by the LSD test in SPSS (*p* < 0.05) [50]. The nucleotide diversity (Pi) values were analyzed by the sliding window algorithm in DnaSP v6.12.03 (Barcelona, Spain) with 200 bp-sliding windows and 20 bp-step sizes [49].

### 3.4. Repeat Elements Analysis

The online MISA software detected the simple sequence repeats (SSRs) of the *R. nymphaeae* mitogenome (http://pgrc.ipk-gatersleben.de/misa/misa.html, accessed on 30 July 2024). The searching parameters of the minimum numbers of the monomeric, dimeric, trimeric, tetrameric, pentameric, and hexameric nucleotides were set as 10, 5, 4, 3, 3 and 3, respectively. Meanwhile, the dispersed repeats (≥30 bp) in the mitochondrial genome were detected via REPuter online tool (https://bibiserv.cebitec.uni-bielefeld.de/reputer/, accessed on 30 July 2024). The results are shown into four types: forward, reverse, palindromic, and complementary repeats.

### 3.5. Phylogenetic Analysis

Complete and partial mitochondrial genomes of 41 aphid species were retrieved and obtained from GenBank (https://www.ncbi.nlm.nih.gov/genbank/, accessed on 19 June 2024); information concerning these genomes is shown in Table S4. Two rRNAs and thirteen PCGs were extracted and then used for phylogenetic analysis [48]. The PCGs nucleotide sequences were aligned by MAFFT codon alignment, and MAFFT aligned the rRNA sequences with the L-INS-I strategy. Gblocks were used to remove any gaps and ambiguously aligned sites after the alignment [51]. These aligned genes (13 PCGs + 2 rRNAs) were then concatenated. ModelFinder was used to evaluate the optimal partitioning scheme and substitution models on PhyloSuite v1.2.3 with linked branch lengths, BIC and searching by the greedy algorithm. The best-fit mode GTR+F+I+G4 model was used in the subsequent phylogenetic analysis. The maximum likelihood of the IQ tree was determined using 1000 bootstraps. Bayesian inference was performed in MrBayes3.2.7 based on the MCMC (Markov Chain Monte Carlo) algorithm with four chains. Two independent runs of 2,000,000 generations were conducted, and the trees were sampled every 100 generations to obtain the average deviation of the split frequencies falling below 0.01. The first 25% of trees were discarded as burn-in [52].

## 4. Conclusions

The complete mitogenome of *Rhopalosiphum nymphaeae* was sequenced and its characteristics were described in this study. This mitochondrial genome was 15,772 bp in length and had a high A+T content in 13 PCGs, 22 tRNAs, 2 rRNAs, the control region and the repeat region. The nucleotide composition, gene order, and codon usage had high conservation in Aphidinae. ENC-, PR2- and neutrality plot analysis revealed that the PCGs of *R. nymphaeae* were under strong natural selection. The evolutionary rates of all 13 mitochondrial PCGs indicated that they had experienced purifying selection. The phylogenetic analysis supported the monophyly of Aphidinae and Rhopalosiphina. Although the data in this study could not resolve the monophyly of *Rhopalosiphum*, it was helpful in providing more mitochondrial genome characteristics and phylogenetic information to assist us in understanding the evolution and phylogenetic relationship of aphids.

## Figures and Tables

**Table 1:** Annotation and gene organization of the *Rhopalosiphum nymphaeae* mitochondrial genome.

Gene	Strand	Position	Size (bp)	Start Codon	Stop Codon	Anticodon	Intergenic
COXI	+	1–1531	1531	ATA	T		1
tRNA-Leu2	+	1532–1599	68			UAA	0
COX2	+	1603–2274	672	ATA	TAA		3
tRNA-Lys	+	2277–2349	73			CUU	2
tRNA-Asp	+	2350–2412	63			GUC	0
ATP8	+	2413–2571	159	ATT	TAA		0
ATP6	+	2552–3205	654	ATT	TAA		−20
COX3	+	3205–3990	786	ATG	TAA		−1
tRNA-Gly	+	3990–4052	63			UCC	−1
NAD3	+	4053–4406	354	ATT	TAA		0
tRNA-Ala	+	4406–4469	64			UGC	−1
tRNA-Arg	+	4469–4534	66			UCG	−1
tRNA-Asn	+	4534–4600	67			GUU	−1
tRNA-Ser1	+	4600–4661	62			GCU	−1
tRNA-Glu	+	4664–4729	66			UUC	2
RR	+	4730–4922	193				0
tRNA-Phe	−	4923–4987	65			GAA	0
NAD5	−	4988–6658	1671	ATT	TAA		0
tRNA-His	−	6711–6774	64			GUG	52
NAD4	−	6775–8083	1309	ATG	T		0
NAD4L	−	8092–8382	291	ATA	TAA		8
tRNA-Thr	+	8384–8445	62			UGU	1
tRNA-Pro	−	8448–8517	70			UGG	2
NAD6	+	8519–9013	495	ATT	TAA		1
CYTB	+	9013–10128	1116	ATG	TAG		–1
tRNA-Ser2	+	10,127–10,191	65			UGA	–2
NAD1	−	10,202–11,137	936	ATT	TAA		10
tRNA-Leu1	−	11,138–11,202	65			UAG	0
rrnaL	−	11,202–12,457	1256				−1
tRNA-Val	−	12,458–12,519	62			UAC	0
rrnaS	−	12,532–13,298	767				12
CR	+	13,299–14,407	1109				0
tRNA-Ile	+	14,408–14,471	64			GAU	0
tRNA-Gln	−	14,469–14,534	66			UUG	−3
tRNA-Met	+	14,539–14,604	66			CAU	4
NAD2	+	14,605–15,582	978	ATA	TAA		0
tRNA-Trp	+	15,581–15,642	62			UCA	−2
tRNA-Cys	−	15,635–15,702	68			GCA	−8
tRNA-Tyr	−	15,705–15,771	67			GUA	2

**Table 2:** Nucleotide composition of the mitochondrial genome of *Rhopalosiphum nymphaeae*.

Region	Length	A%	T%	G%	C%	AT%	GC%	AT-Skew	GC-Skew
Fulll ength	15,772	44.98	39.36	5.78	9.88	84.34	15.66	0.067	0.262
PCGs	10,943	35.23	48.41	7.9	8.46	83.64	16.36	−0.158	−0.034
rrnaL	1256	47.13	38.06	4.62	10.19	85.19	14.81	0.106	−0.376
rrnaS	767	43.02	40.68	5.74	10.56	83.70	16.30	0.028	−0.296
tRNAs	1438	44.23	41.38	8.48	5.91	85.61	14.39	0.033	0.179
CR	1109	44.27	43.82	3.34	8.57	88.09	11.91	0.005	−0.439
RR	193	50.78	35.23	4.15	9.84	86.01	13.99	0.181	−0.407
+ strand	7632	38.92	43.78	7.02	10.29	82.7	17.31	0.059	−0.189
− strand	6829	51.66	33.86	4.83	9.65	85.52	14.48	0.208	−0.333

Note: CR, control region; RR, repeat region.

## Data Availability

The original contributions presented in the study are included in the article/Supplementary Materials; further inquiries can be directed to the corresponding author/s.

## Supplementary Materials

The following supporting information can be downloaded at https://www.mdpi.com/article/10.3390/ijms252111336/s1.
