# Supplementary material for: Characterization, Codon Usage Pattern and Phylogenetic Implications of the Waterlily Aphid Rhopalosiphum nymphaeae (Hemiptera: Aphididae) Mitochondrial Genome
Source: Int J Mol Sci. 2024 Oct 22;25(21):11336. doi: 10.3390/ijms252111336 (PMC11547030; doi:10.3390/ijms252111336)
Supplement: Supplementary file 1 [file ijms-25-11336-s001.zip › Table S2 .pdf]

Table S2. Dispersed repeats (>= 30 bp) identified in the mitogenomes of *R. nymphaeae*.

Nr.	Location	Start	End	Location	Start	End	Length	Repeat type	Mismatch base	E-value
1	Control Region	13357	13498	Control Region	13592	13733	142	F	0	1.85E-220
2	Control Region	13357	13498	Control Region	13827	13968	142	F	0	1.85E-220
3	Control Region	13499	13591	Control Region	13734	13826	93	F	0	1.85E-220
4	Control Region	14108	14143	Control Region	14109	14143	36	C	3	2.86E-09
5	NAD2	14822	14853	NAD2	14834	14865	32	C	2	1.69E-08
6	NAD2	15265	15296	NAD2	15265	15296	32	R	2	1.67E-08
7	rrnL	12024	12054	rrnaL	12024	12054	31	R	2	6.35E-08
8	COX3	3647	3677	COX3	15508	15538	31	R	2	6.28E-08
10	ATP8	2494	2523	ATP8	12104	12133	31	C	2	2.35E-07
11	COX1	1423	1453	COXC1	11208	11238	31	C	3	1.82E-06
12	NAD3	4090	4120	NAD3	4096	4126	31	R	3	1.82E-06
13	NAD3	4304	4334	NAD3	7595	7625	31	P	3	1.82E-06
14	rrnS	13149	13179	Control Region	13519	13549	31	R	3	1.82E-06
15	rrnS	13149	13179	Control Region	13754	13784	31	R	3	1.82E-06
16	NAD4	7789	7818	NAD4	7789	7818	30	R	2	2.35E-07
17	NAD6	8779	8808	NAD6	8779	8808	30	P	2	2.35E-07
18	rrnaL	11954	11983	rrnaL	15456	15485	30	C	2	2.35E-07
19	Control Region	13993	14022	Control Region	13993	14022	30	P	2	2.35E-07
