# Supplementary material for: Characterization, Codon Usage Pattern and Phylogenetic Implications of the Waterlily Aphid Rhopalosiphum nymphaeae (Hemiptera: Aphididae) Mitochondrial Genome
Source: Int J Mol Sci. 2024 Oct 22;25(21):11336. doi: 10.3390/ijms252111336 (PMC11547030; doi:10.3390/ijms252111336)
Supplement: Supplementary file 1 [file ijms-25-11336-s001.zip › Table S3.pdf]

Table S3 SSRs identified in the mitochondrial genome of *Rhopalosiphum nymphaeae*

SSR nr.	SSR type	SSR	Size	Start	End
1	p1	(A)11	11	5231	5241
2	p1	(A)24	24	5562	5585
3	p1	(A)11	11	6582	6592
4	p1	(A)16	16	6642	6657
5	p1	(A)37	37	6663	6699
6	p1	(A)27	27	7992	8018
7	p1	(A)10	10	10584	10593
8	p1	(A)11	11	10595	10606
9	p1	(T)13	13	14255	14267
10	p1	(T)10	10	14326	14335
11	p1	(T)10	10	15276	15285
12	p2	(AT)5	10	8457	8466
13	p2	(TA)9	18	13998	14015
14	p2	(AT)8	16	14058	14073
15	p2	(AT)10	20	14117	14136
16	p2	(TA)6	12	14169	14180
17	p3	(TAA)4	12	317	328
18	p3	(ATT)4	12	3098	3109
19	p3	(TAA)4	12	3660	3671
20	p3	(TTA)4	12	9349	9360
21	p4	(ATAA)4	16	7664	7679
22	p4	(AAAT)3	12	8367	8378
23	p4	(AAAT)3	12	11949	11960
24	p4	(TAAA)3	12	12033	12044
25	p4	(TAAA)3	12	12396	12407
26	p4	(AAAT)3	12	12639	12650
27	p4	(AATT)3	12	13588	13599
28	p4	(AATT)3	12	13823	13834
29	p4	(TTAT)4	16	15564	15579
30	p5	(ATAAA)3	15	7763	7777
31	p6	(ATTAAT)3	18	8782	8799
32	p6	(ATAAAA)3	18	10452	10469
33	p6	(AAAATT)3	18	11589	11606
