# Supplementary material for: Characterization, Codon Usage Pattern and Phylogenetic Implications of the Waterlily Aphid Rhopalosiphum nymphaeae (Hemiptera: Aphididae) Mitochondrial Genome
Source: Int J Mol Sci. 2024 Oct 22;25(21):11336. doi: 10.3390/ijms252111336 (PMC11547030; doi:10.3390/ijms252111336)
Supplement: Supplementary file 1 [file ijms-25-11336-s001.zip › Table S4.pdf]

Table S4 mitochondrial genome information used in this study

ID	Taxon	Accession number	Size(bp)
1	*Acyrthosiphon pisum*	NC_011594.1	16971
2	*Adelges laricis*	KP722589.1	12146
3	*Aiceona himalaica*	KP722590.1	9991
4	*Aphis aurantii*	MN871977.1	15296
5	*Aphis citricidus*	MK540501.1	16763
6	*Aphis craccivora*	KX447142.1	15305
7	*Aphis fabae mordvilkoi*	NC_039988.1	15346
8	*Aphis glycines*	MK111111.1	17954
9	*Aphis gossypii*	NC_024581.1	15869
10	*Aphis spiraecola*	MN316642.1	15465
11	*Baizongia pistaciae*	NC_035314.1	15602
12	*Cavariella salicicola*	NC_022682.1	16317
13	*Ceratovacuna lanigera*	KP722586.1	11549
14	*Cervaphis quercus*	NC_024926.1	15272
15	*Daktulosphaira vitifoliae*	DQ021446.1	12349
16	*Diuraphis noxia*	NC_022727.1	15784
17	*Eriosoma lanigerum*	NC_033352.1	15640
18	*Eucallipterus tiliae*	KP722573.1	12146
19	*Floraphis choui*	NC_035310.1	15308
20	*Floraphis meitanensis*	NC_035316.1	15301
21	*Greenidea kuwanai*	KP722580.1	12146
22	*Hormaphis betulae*	NC_029495.1	15088
23	*Kaburagia rhusicola ovatirhusicola*	MF043985.1	16184
24	*Kaburagia rhusicola rhusicola*	MF043987.1	16159
25	*Schlechtendalia elongallis*	NC_035315.1	16191
26	*Schlechtendalia flavogallis*	NC_035312.1	16150
27	*Melaphis rhois*	NC_036065.1	15436
28	*Myzus persicae*	NC_029727.1	17382
29	*Neothoracaphis yanonis*	KP722574.1	12146
30	*Nurudea ibofushi*	NC_035311.1	16054
31	*Nurudea shiraii*	NC_035301.1	15389
32	*Nurudea yanoniella*	NC_035313.1	15858
33	*Pterocomma pilosum*	KC840676.1	12529
34	*Rhopalosiphum nymphaeae Hunan*	MZ420705.1	15302
35	*Rhopalosiphum nymphaeae Korea*	NC_046740.1	15594
36	*Rhopalosiphum padi*	KT447631.1	15205
37	*Rhopalosiphum rufiabdominalis*	NC_062327.1	15289
38	*Schizaphis graminum*	NC_006158.1	15721
39	*Schlechtendalia chinensis*	NC_032386.1	16047
40	*Schlechtendalia peitan*	NC_035302.1	15609
41	*Sitobion avenae*	NC_024683.1	15180
42	*Tuberolachnus salignus*	KP722566.1	11162
