# Supplementary figures and images for: Characterization, Codon Usage Pattern and Phylogenetic Implications of the Waterlily Aphid Rhopalosiphum nymphaeae (Hemiptera: Aphididae) Mitochondrial Genome
Source: Int J Mol Sci. 2024 Oct 22;25(21):11336. doi: 10.3390/ijms252111336 (PMC11547030; doi:10.3390/ijms252111336)

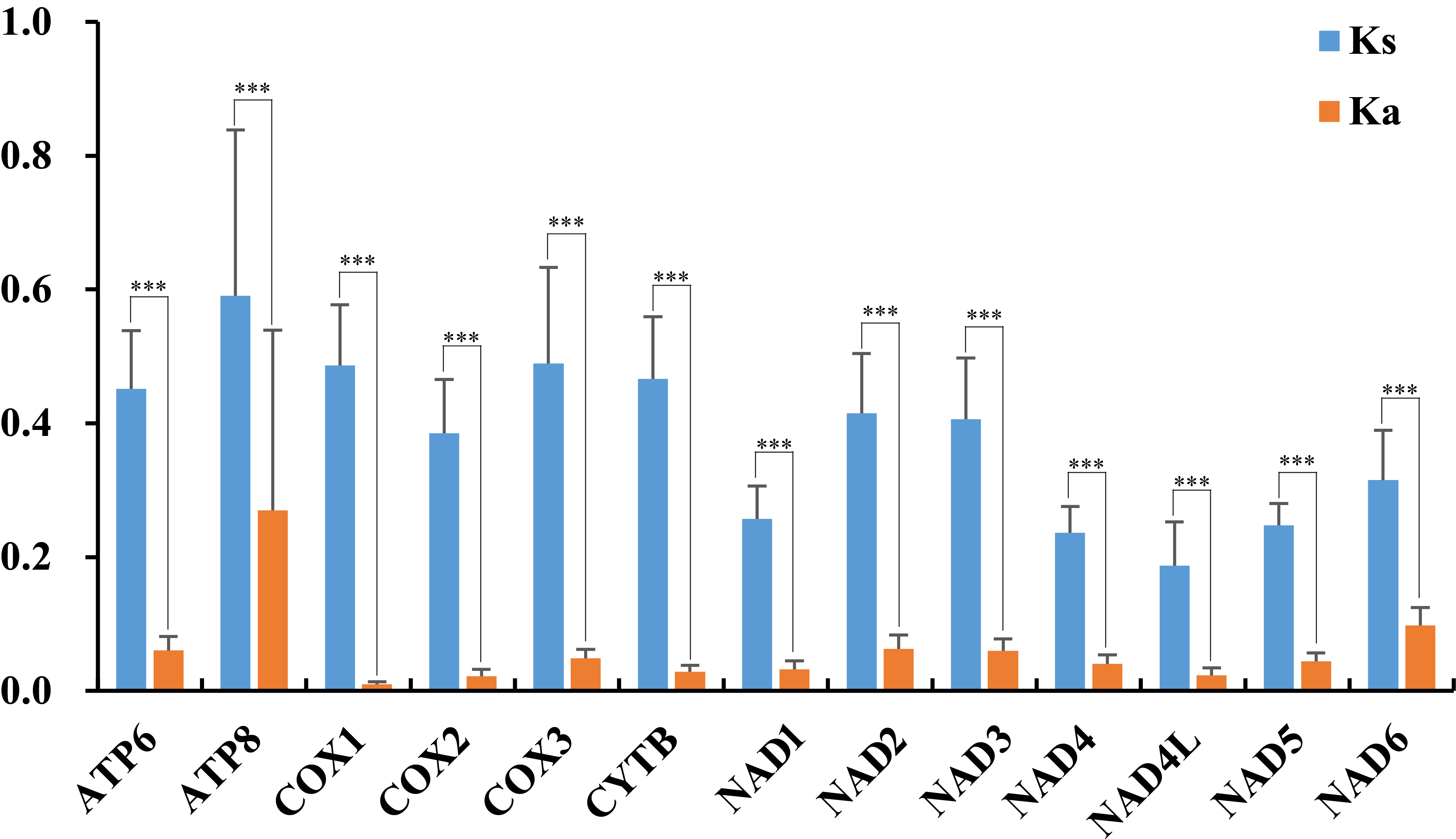

Supplement: Supplementary file 1 [file ijms-25-11336-s001.zip › Figure S1.tif]

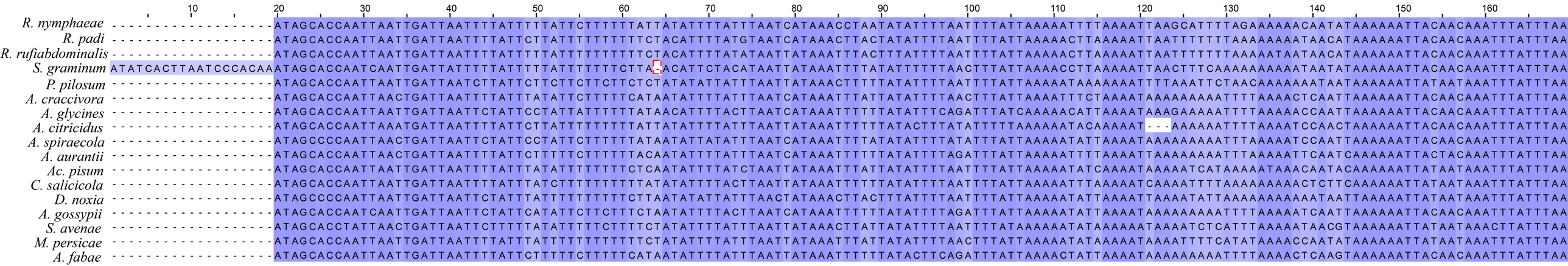

Supplement: Supplementary file 1 [file ijms-25-11336-s001.zip › Figure S2.tif]

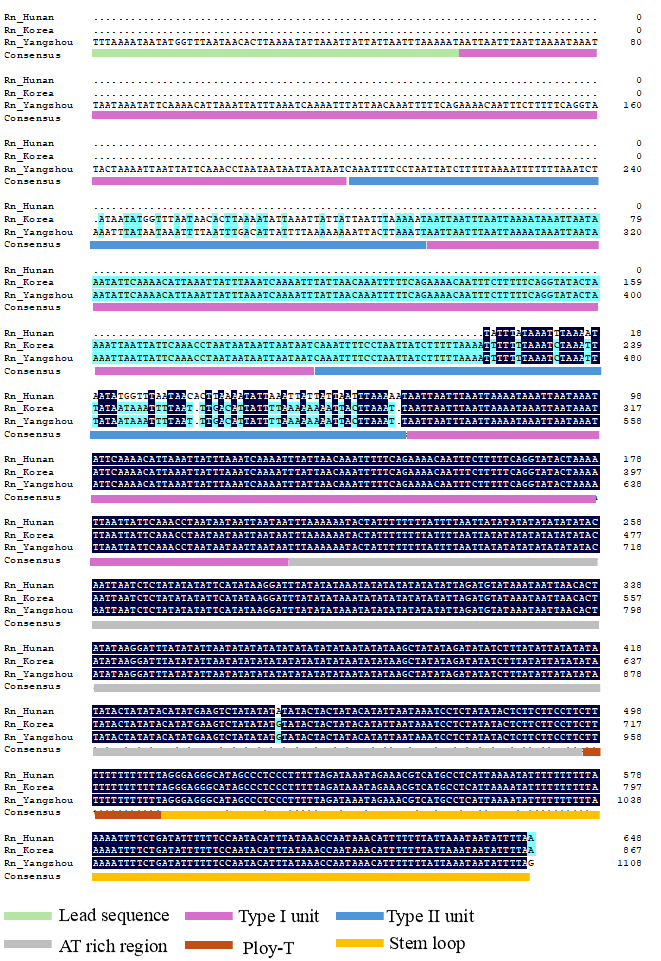

Supplement: Supplementary file 1 [file ijms-25-11336-s001.zip › Figure S3.tif]
